# P-1644. Antimicrobial Stewardship Opportunities for Urine Cultures Resulting After Patient Discharge

**DOI:** 10.1093/ofid/ofae631.1810

**Published:** 2025-01-29

**Authors:** Natalie Harris, Jenny Shroba, Joshua Burrows, Connor R Deri, Alaattin Erkanli, Jason Funaro, Hui-Jie Lee, Rebekah W Moehring, Michael E Yarrington, Rebekah Wrenn

**Affiliations:** Duke University Hospital, Durham, North Carolina; Duke University Hospital, Durham, North Carolina; Duke University Hospital, Durham, North Carolina; Duke University, Durham, North Carolina; Duke University Hospital, Durham, North Carolina; Duke University Hospital, Durham, North Carolina; Duke University Hospital, Durham, North Carolina; Duke University, Durham, North Carolina; Duke University Health System, Durham, North Carolina; Duke University, Durham, North Carolina

## Abstract

**Background:**

Urinary tract infections (UTIs) constitute 3 million emergency department visits (ED) annually and 15% of outpatient antibiotic prescriptions. The outpatient setting is the highest volume of antimicrobial prescribing in the United States, however, is generally a neglected practice area for antimicrobial stewardship (AS) programs. This study sought to evaluate need for AS with an existing ED feedback system for ambulatory patients.

Primary Outcome

**Figure:**
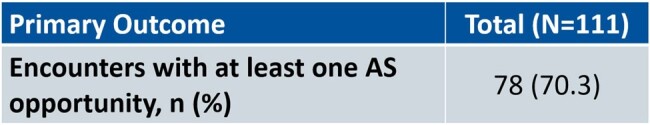

**Methods:**

This multi-center, retrospective study included adults > 18 years old and admitted inpatient or visited the emergency department from August 1, 2022 to February 1, 2023. Patients were included if prescribed antibiotics at discharge or within 72 hours of discharge and had a significant update to urine culture results post discharge up to 14 days. Patients were excluded if prescribed non-urinary indication antibiotic, deceased prior to final culture result, or discharged to hospice. A random 10% convenience sample was analyzed. Descriptive statistics were used to summarize patient characteristics and primary and secondary objectives.

Secondary Outcomes

**Figure:**
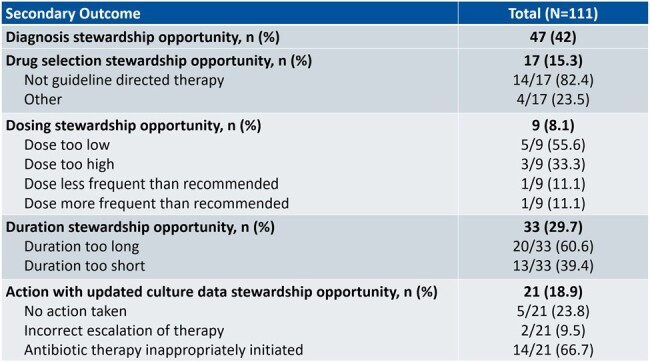

**Results:**

From a cohort of 1,362 patients, 137 encounters were screened and 111 met the inclusion criteria. Over 70% of encounters had at least one AS opportunity. Misdiagnosis attributed to 42% of all opportunities for intervention. Additionally, 20 (18%) patients received longer duration of antibiotics than indicated, and upon culture review, 14 (12.6%) patients received antibiotics post-discharge despite initial diagnosis of asymptomatic bacteriuria.

**Conclusion:**

The majority of patients prescribed antibiotics with pending urine culture data receive unnecessary exposure in our system. Given the high frequency of misdiagnosis of UTI’s and errors in antibiotic prescribing found in this cohort, it is ripe for AS intervention and is uniquely structured with existing culture review systems as present in many EDs. Provision of decision support and provider education to reduce risks of unnecessary antibiotic therapy is needed.

**Disclosures:**

**Rebekah W. Moehring, MD, MPH, FIDSA, FSHEA**, UpToDate, Inc.: Author Royalties

